# THE ROLE OF HEALTH LITERACY IN HEALTH CARE UTILIZATION AMONG TAIWANESE OLDER ADULTS

**DOI:** 10.1093/geroni/igad104.3028

**Published:** 2023-12-21

**Authors:** Zih-Yong Liao, Pei-Chun Liao, Fang-Lin Kuo

**Affiliations:** National Health Research Institutes, Yunlin, Yunlin, Taiwan (Republic of China); National Health Research Institutes, Yunlin, Yunlin, Taiwan (Republic of China); National Health Research Institutes, Yunlin, Yunlin, Taiwan (Republic of China)

## Abstract

Health literacy is one of important capabilities links to health-related behaviours. The extant study provides limited information about the healthcare access of older adults. This study investigated factors determining the healthcare utilisation of 2423 community-dwelling older adults from the Taiwan Longitudinal Study on Ageing between 2011 and 2015. We used linear mixed-effects regression models and cumulative logit models to examine whether personal characteristics are associated with healthcare utilisation. A significant association was found between the level of difficulties in health literacy and healthcare utilisation after controlling for age, comorbidity, depression, mobility and accessibilities. The higher the older adults scored in the difficulty of health literacy, the lower frequencies of clinics (p = .03), inpatient (p = .02) and dentist visits (p = .02). Individuals who access health-related information more easily have a better chance to receive inpatient services when they feel unwell (p = .0004). Better perceived health is less likely to use clinics (p < .0001), emergency (p < .0001) and inpatient services (p = .0007) but not include visits to pharmacy and traditional medicine. People engaging in workouts are less likely to purchase in a pharmacy (p = .02) but visit traditional medicine (p = .02) more often. It echoes that health literacy is an intermediate attribute to self-efficacy in managing personal health matters and suggests a perspective that older adults consider taking traditional medicine as a self-care instead of healthcare treatment. Future intervention programmes can address health literacy promotion as an initiating step to facilitate participants’ engagement.

